# Supplementary material for: Content-rich biological network constructed by mining PubMed abstracts
Source: BMC Bioinformatics. 2004 Oct 8;5:147. doi: 10.1186/1471-2105-5-147 (PMC528731; doi:10.1186/1471-2105-5-147)
Supplement: Additional File 2 — The original results of the above study (non-essential files are deleted to keep the file size under the limit set by BMC bioinformatics). [file 1471-2105-5-147-S2.bz2 › chilibotAdditionalFile2/dip20/366ID9045692E6040/html/FGF1_FGFR3.html]

 


 **FGF1** and **FGFR3** 
  
Found 170 abstracts in PubMed, retrieved 20.  
 

 What does Google say? 
 PDF only 
| .edu only 

---

**Interactive relationship** (e.g. stimulation, inhibition, etc)

**Neutral relationship**- 2 to determine the distribution, in the mature mammalian cochlea, of FGF 1  [ **FGF1** ]  and the receptor,  **FGFR3** , to which it binds with high affinity.  Ref: 12512896 J Otolaryngol, 2002
- The findings show that the surgically created lesions contain only a small amount of FGF  [ **FGF1** ]  receptor 3  **FGFR3**  expressed on mesenchymal stem cells located in the perichondrium, as compared to the cell population carrying  **FGFR3**  in the contralateral limb.  Ref: 12645631 Exp Mol Pathol, 2003
- Importantly, we also find a number of gene expression changes indicating that FGF  [ **FGF1** ]  promotes many aspects of hypertrophic differentiation, a notion supported by in situ analysis of developing growth plates from mice expressing an activated form of  **FGFR3** .  Ref: 12821644 J Cell Biol< MedlineTA>J Cell Biol, 2003

**Non-interactive relationship** (e.g. studied together, co-existance, homology, etc.)

- Since the presence of FGF  [ **FGF1** ]  receptors FGFRs determines cellular responsiveness, we examined the localisation of FGFR1, FGFR2 and  **FGFR3**  expression by immunohistochemistry throughout the repair of full thickness excisional wounds up to 28 days after wounding.  Ref: 12648559 Arch Oral Biol, 2003
- RESULTS A large stromal subpopulation expresses fibroblast growth factor receptor 3  [ **FGFR3** ]  FGF  [ **FGF1** ]  R3, indicating a mesenchymal origin of these cells.  Ref: 12865629 PathobiologyPathobiology, 2003
- Immunoreactivity of both FGF 1  [ **FGF1** ]  and  **FGFR3**  was observed in spiral ganglion neurons, inner and outer hair cells, pillar cells, and Dieter and Hensen s cells.  Ref: 12512896 J Otolaryngol, 2002
- In complementary in vitro assays, bFGF did not directly stimulate avian OC development from bone marrow mononuclear cell precursors, consistent with their low mRNA expression of the four avian signaling FGF  [ **FGF1** ]  receptors FGFR 1, FGFR 2, FGFR 3  [ **FGFR3** ] , and FGFR like embryonic kinase FREK.  Ref: 12369790 J Bone Miner Res, 2002
- Immunoreactivity for receptors R for IGF 1 and FGF  [ **FGF1** ]  2 IGF 1R, FGFR1, and  **FGFR3**  was most pronounced in chondroblasts and hypertrophic chondrocytes, while FGFR2 immunoreactivity was strongest in the articular and prechondroblastic zones.  Ref: 12243968 Arch Oral Biol, 2002
- The effects of increased  **FGFR3**  activation were determined by exposing cochlear explants to FGF2, a strong ligand for several FGF  [ **FGF1** ]  receptors.  Ref: 12417662 J Neurosci, 2002
- Standard in situ immunohistochemical techniques were used to stain each cochlea for FGF 1  [ **FGF1** ]  and  **FGFR3** , apoptotic markers p53 and p21, Bcl 2, and the cell cycling marker proliferating cell nuclear antigen PCNA.  Ref: 12512896 J Otolaryngol, 2002
- Fibroblast growth factor receptor 3  [ **FGFR3** ]   **FGFR3**  is one of the membrane bound tyrosine kinases that mediate the actions of fibroblast growth factor FGF  [ **FGF1** ]  family members.  Ref: 12687892 Pediatr Pathol Mol Med< MedlineTA>Pediatr Pathol Mol Med, 2002
- Using semiquantitative reverse transcription polymerase chain reaction, we examined the expression of insulin like growth factor 1 Igf 1, fibroblast growth factor 2 Fgf  [ **FGF1** ]  2, and their receptors Igf 1r, Fgfr1, Fgfr2, and  **Fgfr3**  in the MCC of 28 day old rats at, and 14 days after placing intraoral appliances designed to produce a lateral functional shift of the mandible.  Ref: 12594422 Am J Orthod Dentofacial Orthop, 2003
- Activating mutations in FGF  [ **FGF1** ]  receptor 3  **FGFR3**  cause several human dwarfism syndromes by affecting both chondrocyte proliferation and differentiation.  Ref: 12821644 J Cell Biol< MedlineTA>J Cell Biol, 2003
